# Dual-network hydrogel loaded with antler stem cells conditioned medium and EGCG promotes diabetic wound healing through antibacterial, antioxidant, anti-inflammatory, and angiogenesis

**DOI:** 10.1016/j.mtbio.2025.101612

**Published:** 2025-02-24

**Authors:** Xianyi He, Yichen Gao, Xia Wang, Chuankai Zhang, Zhaoxin Xia, Wei Xu, Hua Yang, Gang Tao, Rui Cai, Junliang Chen, Yun He

**Affiliations:** a Luzhou Key Laboratory of Oral & Maxillofacial Reconstruction and Regeneration, The Affiliated Stomatological Hospital, Southwest Medical University, Luzhou, 646000, China; b Department of Oral and Maxillofacial Surgery, The Affiliated Stomatological Hospital, Southwest Medical University, Luzhou, 646000, China; c Institute of Stomatology, Southwest Medical University, Luzhou, 646000, China; d Department of General Dentistry, The Affiliated Stomatological Hospital, Southwest Medical University, Luzhou, 646000, China; e Department of Oral and Maxillofacial Surgery, The Deyang Stomatological Hospital, Deyang, 618000, China

**Keywords:** Diabetic wound healing, Antler stem cells, Conditioned medium, Angiogenesis, Hydrogel

## Abstract

Diabetic wound healing is characterized by persistent inflammation, reactive oxygen species overproduction, bacterial infection, and compromised angiogenesis. In recent years, Antler Stem Cells (ASCs) have attracted attention because of their potential role in promoting wound healing by promoting cell proliferation and angiogenesis via paracrine effects. In addition, epigallocatechin gallate (EGCG), the main component of green tea, exhibits antibacterial, anti-inflammatory, and antioxidant properties. In this study, we designed and fabricated a gelatin (G)/sodium alginate (SA)-based (SA/G) dual-network hydrogel loaded with ASC-derived conditioned medium (ASC-CM) and EGCG (CEGA) that exhibited excellent swelling capacity, sustained release, and mechanical properties. Both in vitro and in vivo experiments demonstrated that CEGA hydrogels were capable of enhancing cell proliferation, promoting angiogenesis, exhibiting antibacterial properties, mitigating inflammation, and regulating macrophage polarization. These results substantiate their potential application as novel dressings for healing diabetic skin wounds.

## Graphical abstract

**Figure:**
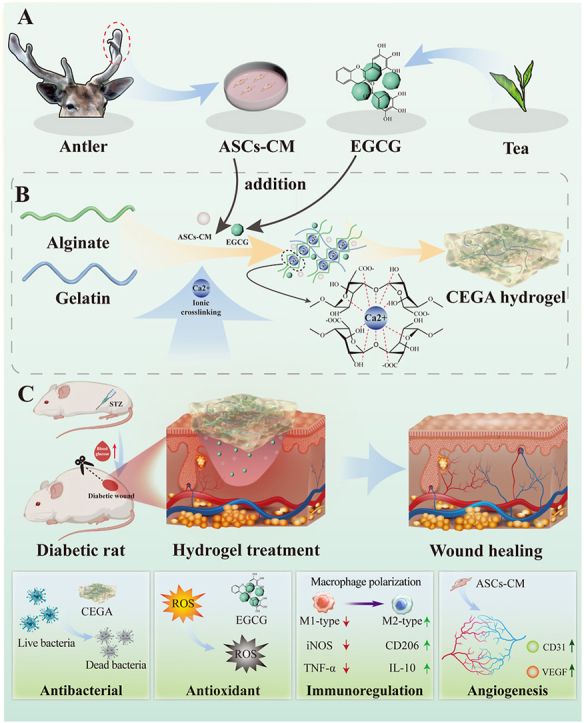

## 1 Introduction

The prevalence of diabetes has been steadily increasing over the past few decades and is estimated to affect approximately 439 million individuals by 2030 [1]. Diabetic wounds are characterized by persistent inflammation, overproduction of reactive oxygen species, bacterial infection, polarization, and dysregulation of macrophages and abnormal angiogenesis, and about 50–70 % of limb amputations are due to diabetic wounds [[2], [3], [4]]. Conventional diabetic wound management predominantly focuses on late-stage interventions, including bandaging, negative-pressure therapy, hyperbaric oxygen therapy, and skin grafting; however, the therapeutic outcomes remain suboptimal [[5], [6], [7]]. Thus, there is an immediate need to create innovative wound dressings that can effectively facilitate the healing of diabetic wounds at every stage of development.

Recently, stem cells have been found to exhibit remarkable therapeutic effects in facilitating skin remodeling, tissue angiogenesis, soft tissue regeneration, and hair follicle regeneration [8,9]. Antler stem cells (ASCs) are derived from antlers, which are the only known mammalian organs capable of annual regeneration, and are gaining increasing attention because of their remarkable ability to proliferate and repair tissues [10,11]. Researches have shown that ASCs exert their effects primarily through paracrine mechanisms, secreting a plethora of bioactive factors collectively referred to as conditioned medium (CM) [12,13]. CM has the advantages of low immunogenicity, absence of cytotoxicity, and high specificity, and its therapeutic effect on tissue regeneration is comparable to that of ASCs [14]. ASC-CM has been shown to possess substantial potential for promoting bone, cartilage, and vascular regeneration, while also demonstrating anti-inflammatory properties conducive to wound healing, which indicates that ASC-CM-based therapies could represent a promising strategy for managing diabetic wounds [12,[15], [16], [17]]. Some studies have directly injected the active components of these stem cells into or beneath the wound edges, which makes it difficult to effectively retain stem cell components. Therefore, developing a suitable wound dressing capable of carrying ASC-CM has become an important focus of current research [18].

Hydrogels have been widely used as carriers for drug delivery, which not only prolong the action time of cytokines on the wound surface by protecting them from adverse degradation, but also provide a moist environment for wounds because of their high porosity and hydration ability to promote the exchange of nutrients and oxygen [[19], [20], [21]]. Gelatin (G), a collagen derivative with excellent biocompatibility and biodegradability, is widely used in tissue engineering and drug delivery [22]. Studies have shown that the combining gelatin with another biopolymer can overcome the limitations of gelatin in terms of its rigidity and brittleness [23]. Sodium alginate (SA), derived from brown seaweed, is one of the most widely used biomaterials because it is nontoxic, easy to produce, biodegradable, and inexpensive. SA can be incorporated into cross-linked gelatin through ion-exchange interactions and hydrogen bond formation to minimize the gelation time and improve the mechanical properties of the composites [24,25]. Therefore, this study aimed to use a SA/G dual-network hydrogel loaded with ASC-CM as a dressing for treating diabetic wounds.

Hyperglycemia and elevated levels of oxidative stress in diabetic wounds can lead to inflammatory infections and delayed wound healing, which are not conducive to all stages of wound healing [26,27]. The primary polyphenol in green tea, epigallocatechin gallate (EGCG), has antibacterial activity and free radical scavenging properties, and has been shown to reduce the expression of pro-inflammatory factors while increasing the level of anti-inflammatory cytokine interleukin-4 (IL-4) in lipopolysaccharide (LPS) -induced macrophages in vitro [28,29]. Hence, EGCG has the potential for application in the improvement of the diabetic wound microenvironment.

**Fig. 1:**
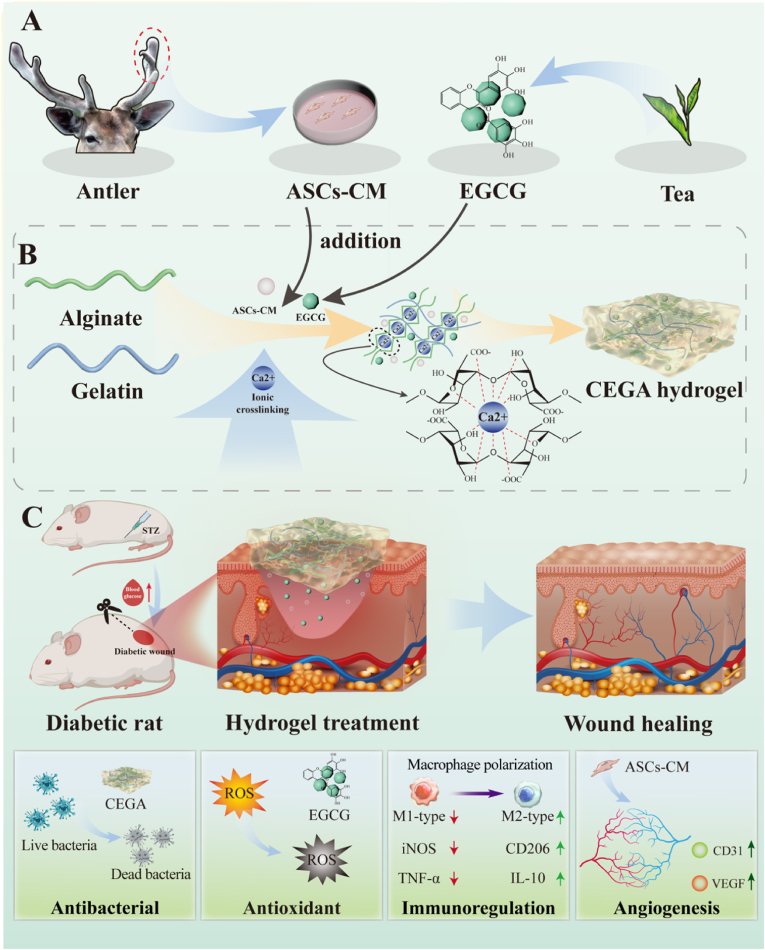
Schematic illustration of preparing and applying CEGA hydrogel with multifunctional properties for promoting diabetic wound healing. (A) Schematic diagram of ASC-CM and EGCG extraction. (B) Preparation of CEGA hydrogel. (C) The antibacterial, antioxidative, immunomodulatory, and angiogenic functions of CEGA in promoting the healing of diabetic wounds.

In this study, we designed and fabricated a gelatin/sodium alginate-based composite hydrogel incorporating ASC-CM and EGCG, named CEGA (Fig. 1), and investigated its potential applications in diabetic wound healing. These results indicate that the CEGA hydrogel has excellent controlled release and mechanical properties. Through the action of ASC-CM, CEGA markedly promoted the proliferation of human umbilical vein endothelial cells (HUVECs), L929 fibroblasts, and mouse mononuclear macrophage leukemia cells (RAW264.7). In addition, CEGA exhibits effective antibacterial, antioxidant, angiogenic, and macrophage polarization-regulating properties both in vitro and in vivo. Overall, CEGA hydrogel may become a potential alternative treatment for diabetic wounds.

## 2 Materials and methods

### 2.1 Materials

Gelatin, sodium alginate, and EGCG were purchased from Macklin Biochemical Co., Ltd. (Shanghai, China). Dulbecco's Modified Eagle's medium (DMEM) and fetal bovine serum (FBS) were purchased from Gibco (Waltham, USA). The Cell Counting Kit-8 (CCK-8) was sourced from Solarbio (Beijing, China). Rats used in this study were obtained from the Experimental Animal Center of Southwest Medical University (Luzhou, China).

### 2.2 Isolation, culture, and identification of ASCs

Antlers were harvested from a 2-year-old healthy deer and the distal 4 cm of the growing tip was obtained. The reserve mesenchyme (RM) layer tissue was cut into 1 mm^3^ fragments for primary cell culture. The detailed procedures for the isolation and identification of primary ASCs have been described by Wang et al. [30]. ASCs were characterized by surface marker profiling (CD29, CD44, CD90, CD105, CD31, and CD45) via flow cytometry (FCM) and by keratin and vimentin via immunohistochemistry staining (IHC). For FCM, the cell suspension was stained with primary antibodies, washed with phosphate-buffered saline (PBS), stained with secondary antibodies, washed again, and quantitatively analyzed using BD FACSCelesta (BD Biosciences, USA).

### 2.3 Preparation of ASC-CM

CM was prepared as previously described. Briefly, ASCs were seeded in six-well plates at a density of 1 × 10^5^ cells/well. Once the cells reached approximately 80 % confluence, the normal medium with 10 % FBS was replaced with serum-free DMEM after three PBS washes. After incubation for 48 h, the supernatants were collected as CM for subsequent experiments.

### 2.4 Preparation of hydrogels

The method described by Sabzevari et al. [31] was modified to prepare SA/G hydrogels. The specific procedure was as follows: firstly, 8 g gelatin powder and 2 g sodium alginate powder were weighed and added to a beaker filled with 85 mL deionized water, and then the gelatin powder and sodium alginate powder were gradually dissolved using a magnetic stirrer at 60 °C and 200 rpm/min. Subsequently, the solution was stirred slowly at 100 rpm to eliminate residual bubbles. Initially, 3 mL of the mixed solution was added to the mold. Once the SA/G solution cooled to approximately 37 °C, 3 mL of either PBS, ASC-CM, PBS containing EGCG, or ASC-CM containing EGCG was added to each respective group, which were labeled as GA, CGA, EGA, and CEGA, respectively. The solution was stirred evenly and placed in a refrigerator at 4 °C for 30 min to allow solidification. The final gelatin and sodium alginate concentrations of the hydrogels were 4 % and 1 %, respectively. Add 6 mL of 2 % CaCl_2_ solution to the surface of the initially solidified hydrogel and soak for 2 h for calcium crosslinking. Finally, the crosslinked hydrogel was taken out of the mold, and the excess CaCl_2_ was rinsed with sterile PBS and stored at 4 °C.

### 2.5 Characterization of hydrogels

Scanning electron microscopy (SEM) images of the hydrogel cross sections were obtained using a scanning electron microscope (VEGA3, TESCAN, China). An accelerating voltage of 10 kV was used. The average pore size was calculated using an image-processing software (ImageJ).

IR spectra were obtained using Fourier Transform Infrared Spectroscopy (WQF-530, Beijing, China). The measurements were conducted at room temperature in the smart ATR (Attenuated Total Reflectance) mode.

This experiment was designed to evaluate the release of EGCG and ASC-CM from CEGA hydrogels. The CEGA hydrogel (5 g) was immersed in 50 mL of PBS and incubated with shaking at 50 rpm. At predetermined time intervals, 100 μL supernatant samples were collected and replaced with an equal volume of fresh PBS. The collected samples were analyzed using a UV spectrophotometer at 274 nm to quantitatively assess the released EGCG. The CEGA hydrogel was immersed in PBS in a 24-well plate, the supernatant was collected every day for eight days. The protein content of the supernatant was determined using BCA Protein Assay Kit (Solarbio, Beijing, China).

Cylindrical dry hydrogels (2 cm in diameter) were immersed in PBS at 25 °C. At specified time intervals, the hydrogels were carefully removed, and any excess liquid on the surface was absorbed using a filter paper. The samples were then weighed, and their weights were recorded until equilibrium swelling was achieved. The swelling ratio (SR) was calculated as follows:

SR(%)=(mt−m0)/m0×100%

where mt is the weight of the swollen hydrogels at each time point and m0 is the weight of the dried hydrogels.

The mechanical properties of each group were assessed using a universal testing machine (INSTRON 5965, Massachusetts, USA). For compression testing, the diameter and height of each hydrogel sample were measured and these parameters were input into the control software for compression at a speed of 10 mm/min. Data on the time, displacement, force, strain, and stress during compression were recorded.

### 2.6 Biocompatibility test of CEGA hydrogel

The biocompatibility of CEGA hydrogel was tested using a hemolytic assay. Fresh blood was drawn from the hearts of four-week-old New Zealand rabbits, anticoagulated with sodium citrate, and diluted in physiological saline for the hemolysis testing. 10 mL of GA, CGA, EGA, and CEGA extracts were incubated for 0.5 h in a shaking water bath at 37 °C. Subsequently, 200 μL of diluted blood was added and homogenized, then maintained at 37 °C for a duration of 60 min. The supernatant was obtained via centrifugation, and the optical density (OD) was measured using a UV–visible spectrophotometer. Negative and positive controls were established by adding 200 μL of diluted blood to 10 mL of PBS or Triton X-100 (1 %). The hemolysis ratio (HR) was calculated as follows:

$$\text{Hemolysis}\;\text{ratio}\;\left(\%\right)=\left[\left({\text{OD}}_{\text{test}}-{\text{OD}}_{\text{nc}}\right)/\left({\text{OD}}_{\text{pc}}-{\text{OD}}_{\text{nc}}\right)\right]\times 100$$

Where OD_test_, OD_pc_, and OD_nc_ are the OD values of the test sample, the positive control, and the negative control, respectively.

The CCK-8 assay and live/dead staining were used to evaluate the biocompatibility of the hydrogel. Ten grams of the hydrogels (GA, CGA, EGA, and CEGA) were immersed in 40 mL of DMEM containing 10 % FBS for 24 h to obtain leachates. L929 fibroblasts, HUVECs, and RAW264.7 were cultured for 24 h in DMEM containing 10 % FBS. The complete medium was subsequently replaced with the leaching solution, and the culture was continued for the 1st, 3rd, and 5th days, during which the assays were performed. Cell viability was measured using a CCK-8 assay kit. Briefly, 10 μL of CCK-8 reagent was added to 100 μL of medium, and the cells were co-cultured for 1 h at 37 °C. The OD value at 450 nm was recorded using a microplate reader (TECAN Infinite M200PRO, China). Additionally, HUVECs and RAW264.7 were cultured with leachates on the 1st, 3rd, and 5th days, respectively. The cells were then incubated with live/dead staining solution for 10 min and observed under a fluorescence microscope (Leica, DMi8, Germany).

### 2.7 In vitro and in vivo antibacterial properties of hydrogel

The antibacterial performances of the hydrogels were evaluated using the bacterial spread plate method. Hydrogels measuring 1 cm in diameter were placed in culture tubes containing 3 mL of bacterial suspension (1 × 10^6^ CFU/mL) comprising *Staphylococcus aureus* (*S. aureus*) and *Escherichia coli* (*E. coli*) and incubated at 37 °C for 8 h. Then, 50 μL of hydrogels-treated bacterial culture medium was spread onto a solidified 2 YT medium. Bacterial growth was observed after culturing at 37 °C for 24 h. Photographs were taken, and colonies in petri dishes were counted. The bacterial suspension was subsequently determined by performing a live/dead survival test based on fluorescence, and the presence of live or dead bacteria was observed under a fluorescence microscope (Olympus BX53, Japan). In the in vivo antibacterial experiment, a bacterial suspension (3 mL, 1 × 10^7^ CFU/mL) was injected into the wounds of 8-week-old adult male Sprague-Dawley (SD) rats and different hydrogel samples were placed within the wound sites. After 1 and 3 d, skin samples were collected and vibrated in 1 mL of PBS for 30 s. Then, 10 μL of the bacterial solution was spread onto a solidified 2 YT medium. After 24 h, photographs were taken, and colonies in the petri dishes were counted.

### 2.8 In vitro antioxidant properties of hydrogel

Reactive oxygen species (ROS) accumulation was assessed using a DCFH-DA radical probe. Initially, RAW264.7 was seeded in a 24-well plate at a density of 1.0–2.0 × 10^5^ cells/well for 24 h, and GA, EGA, CGA, and CEGA hydrogel extracts were added to the cells for an additional 24 h. The Con group was changed with complete medium, and the other groups were changed with complete medium containing 100 μM H_2_O_2_ for 1 h. DCFH-DA, diluted in PBS, was added after removing all the cell culture medium. After incubating at 37 °C for 20 min in the dark, the cells were washed with PBS. An inverted fluorescence microscope was used to visualize the cells.

### 2.9 In vitro scratch assay and tube formation assay

For the scratch assay, HUVECs and L929 fibroblasts were seeded in 6-well plates at a density of 4 × 10^5^ cells/well. After 100 % confluence, a "scratch" without cells was made in the middle of the plate with a 200 μL sterilized pipette tip. Extracts of GA, CGA, EGA, and CEGA hydrogels were added to the corresponding wells, and photographs were taken under an inverted phase microscope to capture the migration of cells at 0 h and 12 h.

The matrix gel was first melted in a refrigerator at 4 °C for 5 h for tube formation assays. Then, 150 μL of the matrix gel was added to a 48-well plate and incubated at 37 °C for 30 min to allow gelation. After gelation, HUVECs were inoculated onto the matrix gel at a density of 3 × 10^4^ cells/well. After incubation for 6 h, cells were photographed under an inverted microscope. The number of junction points was analyzed using the ImageJ software.

### 2.10 Macrophage polarization test

Immunofluorescence staining was used to study the polarization-modulating effect of the CEGA hydrogel on macrophages. RAW264.7 were polarized into M1 and M2 macrophages after 48 h of induction with IL-4 (0.5 μg/mL) and LPS (1 μg/mL), respectively. The IL-4 and LPS groups was treated with IL-4 or LPS alone, respectively. The other material groups were treated with GA, EGA, CGA, and CEGA hydrogel extracts for an additional 48 h. M1 macrophages were stained with the biomarker iNOS, and M2 macrophages were stained with the biomarker CD206 overnight at 4 °C. Images were captured using a confocal microscope (IRX50; Sunny Optical Technology).

### 2.11 In vivo experiments

Type 2 diabetic animal models were established using 8-week-old adult male SD rats (obtained from the Experimental Animal Center of Southwest Medical University). Briefly, the rats were fed high-sugar and high-fat chow (Luzhou Keyang Biotechnology Co., Ltd.) for one month, and then streptozotocin (STZ; 30 mg/kg/day; Cayman Islands, USA) was injected intraperitoneally for five consecutive days. Blood glucose levels were monitored every three days using a glucometer (Accu-Chek Performance, Roche Diagnostics, USA). Rats with fasting blood glucose levels ≥16.7 mM for 1 week were considered as a model of type 2 diabetes [32]. The diabetic rats were divided into six groups: Con, GA, CGA, EGA, and CEGA. After anesthesia, the backs of the rats were shaved and disinfected, and six circular total skin wounds (diameter = 8 mm) were created on the backs of the rats. The wounds were infected with *S. aureus* (50 μL, 1.0 × 10^6^ CFU/mL). Following a 2 h incubation period, different hydrogel samples were placed within the wound sites. Photographic documentation of the wound area was created using a digital camera to observe the progression of wound healing over a specified time period. Wound closure percentage (WC%) was determined using the following formula:

WC(%)=(C0−Ct)/C0×100%

Where C0 is the wound area on the 0th day, and Ct represented the wound area on the 3rd, 6th, 12th, and 18th day.

### 2.12 Histological analysis

On days 6, 12, and 18, tissues surrounding the wound were harvested and fixed using 4 % paraformaldehyde. The obtained tissues were stained with hematoxylin and eosin (H&E) and Masson's trichrome for histological examination. To assess the expression of angiogenesis-related factors and inflammation markers, immunofluorescence staining was used to label CD31, vascular endothelial growth factor (VEGF), CD86, and CD206, while immunohistochemical staining was employed for tumor necrosis factor-alpha (TNF-α) and interleukin-10 (IL-10).

### 2.13 Statistical analysis

All data were presented as means and standard deviations, indicated as the mean ± SD. Comparisons among groups were performed using one-way analysis of variance (ANOVA), followed by Tukey's post-hoc test. The significance was set at∗ *p* < 0.05, ∗∗*p* < 0.01, ∗∗∗*p* < 0.001. When *p* > 0.05, no significant differences were observed (ns).

## 3 Results and discussions

### 3.1 Isolation, culture, and identification of ASCs

**Fig. 2:**
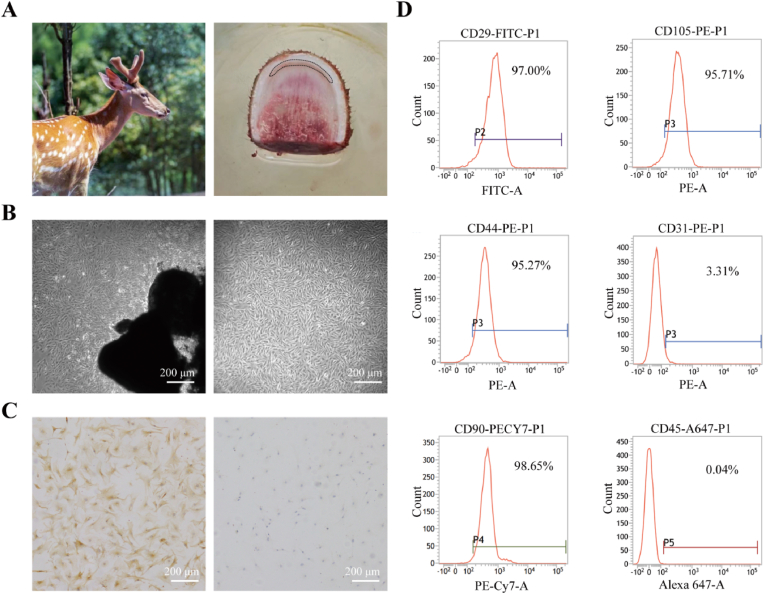
Isolation, culture, and identification of ASCs. (A) ASCs were extracted from the antler mesenchymal layer. (B) Primary and passaged culture of ASCs. (C) IHC staining showed positive expression of vimentin and negative expression of keratin in ASCs. (D) Flow cytometry detected positive expression of ASCs surface antigens CD29, CD44, CD90, and CD105 and negative expression of CD31 and CD45.

ASCs were extracted from the tip of the antler, which is composed of the epidermis, dermis, mesenchymal layer, cartilage layer, and bone layer [33]. The mesenchymal layer in the dotted-line area was collected for this experiment (Fig. 2A). The morphology of the ASCs was homogeneous and fibroblast-like when subcultured until passage three (Fig. 2B). The expression of keratin and vimentin in ASCs were detected by immunohistochemistry. The results showed the positive expression of vimentin and negative expression of keratin, as shown in Fig. 2C. Moreover, according to the flow cytometry data, the cultured ASCs at passage 3 were positive (>95 %) for the surface molecular markers CD29, CD44, CD90, and CD105, and negative (<5 %) for the surface molecular markers CD31 and CD45 (Fig. 2D), which proved that the detected cells were stem cells.

### 3.2 Characterization and physical properties of hydrogels

**Fig. 3:**
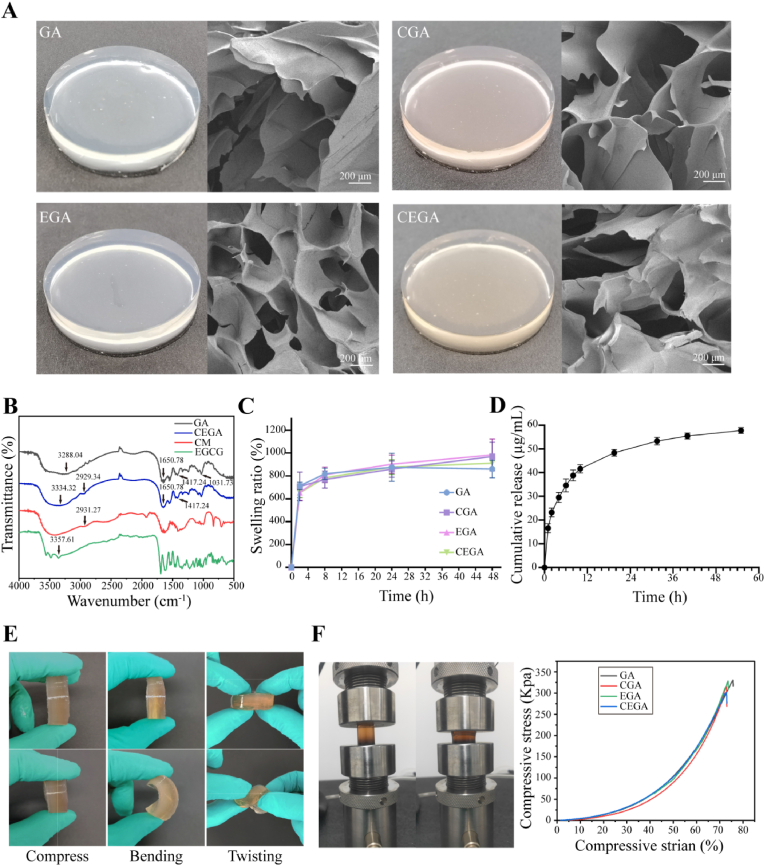
Characterization and physical properties of hydrogels. (A) Visual appearance and SEM images of the following hydrogels: GA, CGA, EGA, and CEGA. (B) FTIR spectra of ASC-CM, EGCG, GA and CEGA. (C) Swelling ratio of hydrogel in each group. (D) Cumulative release properties of CEGA hydrogel for EGCG. (E) Mechanical strength experiment of CEGA hydrogel. (F) Test of mechanical properties of hydrogels in each group.

Recently, hydrogels have garnered significant attention because of their ability to facilitate the slow release of proteins and protect them from degradation [34]. In this study, we developed GA hydrogels encapsulating ASC-CM and EGCG for treating diabetic wounds. At room temperature, the GA and EGA hydrogels appeared colorless and transparent, whereas the incorporation of ASC-CM imparted an orange hue to the CGA and CEGA hydrogels (Fig. 3A). All the hydrogels exhibited minimal shrinkage after freeze-drying. Microstructural analysis of the lyophilized gels revealed the presence of large pores and voids spaces that were uniformly distributed, as shown in Fig. 3A. The GA group exhibited a loose porous mesh-like structure that was not affected by the addition of CM or EGCG alone or together. The porous structure of the hydrogel facilitates the release of payloads and provides sufficient space for foreign functional factors to facilitate the infiltration of new blood vessels, exchange of nutrients, and cell adhesion and proliferation [35].

To elucidate the functional groups in the sponges, the FTIR spectra of the CM, EGCG, and GA, and CEGA sponges, were examined (Fig. 3B). The characteristic bands of the GA hydrogel were 3288.04 cm^−1^, 1650.78 cm^−1^, 1417.24 cm^−1^ and 1031.73 cm^−1^. The peak at 3334.32 cm^−1^ wave number in the CEGA hydrogels containing EGCG may be related to O-H stretching and intramolecular/intermolecular hydrogen bonding [36]. After the addition of EGCG, the stretching frequency shifted to a higher wavelength (3288.04 cm^−1^ and 3334.32 cm^−1^), which is close to the stretching frequency of EGCG (3357.61 cm^−1^). The results showed that EGCG has strong hydroxyl groups in the CEGA hydrogel [37]. The peak values at 1650.78 cm^−1^ and 1417.24 cm^−1^ were attributed to C=O stretching vibration and aromatic ring stretching vibration, respectively. After addition of EGCG, the stretching frequency remained unchanged. This may be because the C=O and aromatic ring stretching groups are common in polysaccharides and phenolic compounds [38]. In addition, with increasing CM content, the peak of the C-H stretching vibration (2929.34 cm^−1^) became broader and clearer, and the amplitude increased. These results showed that EGCG and CM were successfully mixed into the CEGA hydrogel.

In clinical applications, the swelling ability of hydrogel is critically important, and an appropriate swelling capacity is beneficial for the absorption of wound exudates, transport of nutrients and metabolic substances, and the diffusion and release of drugs [39]. The swelling ratio at pH = 7.4 and 37 °C was measured to assess the swelling behavior of the hydrogels. As illustrated in Fig. 3C, all the hydrogels demonstrated a swelling ratio of approximately 700 % at 2 h. Subsequently, the swelling ratios of the hydrogels progressively increased until they reached their respective maxima at 48 h, stabilizing at approximately 900 %. These hydrogels maintained their gel state (stability) at 37 °C throughout the test period, without significant differences in the swelling ratio (*p* > 0.05). The results showed that the hydrogels effectively absorbed wound exudates and avoided exudate accumulation.

The in vitro release profiles of EGCG and ASC-CM from the CEGA hydrogels were recorded to evaluate the suitability of the hydrogels as scaffolds for sustained delivery. As shown in Fig. 3D, CEGA exhibited approximately 42 % release of EGCG at 12 h. At 24 h, the release of EGCG was below the 50 %. Over a 48 h period, the total cumulative percentage release was less than 60 %. These results indicate that the CEGA hydrogel with EGCG content exhibited a higher release rate and prolonged release duration. Moreover, the slow release of EGCG from the CEGA hydrogel may have been due to the coating effect of the hydrogel on the EGCG. The release profile of ASC-CM is shown in Fig. S1. The CEGA hydrogel released ASC-CM at a higher rate in the first two days. The release rate tended to stabilize after 5 days, and the release rate on day 8 was 69.34 ± 4.81 %. In conclusion, our results revealed that the obtained CEGA hydrogel had good controlled release properties for EGCG and ASC-CM with appropriate concentrations.

The increase in mechanical strength plays a crucial role in the stability of the wound dressing to prevent the deformation and collapse of the wound dressing under an external force in the wound area [40]. Therefore, in this study, different types of forces (compression, bending, and twisting) were applied to CEGA hydrogels to verify their material strength. When these forces were maintained for 3 min, none of the hydrogels exhibited any structural damage. After the release, the hydrogel returned to its original state (Fig. 3E). These results indicated that the CEGA hydrogel exhibited good flexibility and resilience.

The compressive strengths of the hydrogels were determined using compression tests. As shown in Fig. 3F, the GA hydrogel fractured at 75 % strain under a compressive stress of 330 kPa. Conversely, the mechanical properties of the CGA/EGA/CEGA hydrogels were slightly inferior; however the maximum compressive strains were still above 70 %, and the compressive stress exceeded 300 kPa. Consequently, benefiting from its high mechanical strength, the CEGA hydrogel appeared to be more reliable in protecting the wound from external stimuli, which could ensure the stability of the wound environment and promote healing.

### 3.3 Biocompatibility evaluation of the hydrogels and secretome release of ASC-CM

**Fig. 4:**
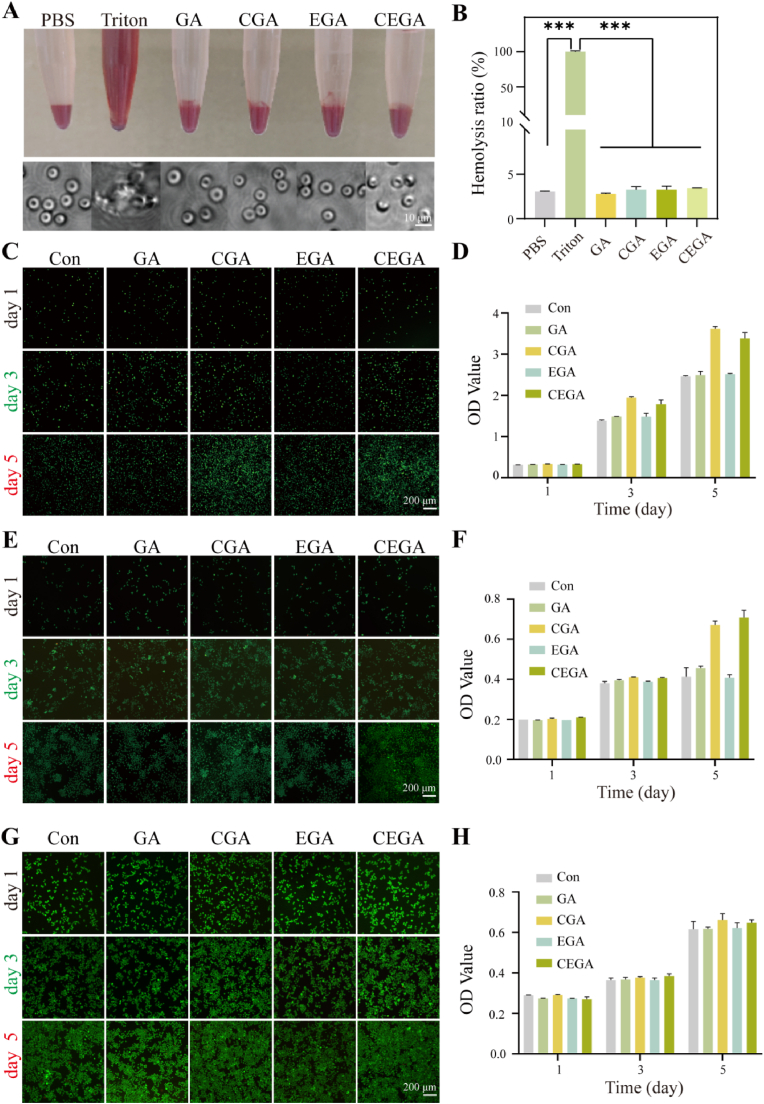
In vitro hemocompatibility and cytocompatibility assays. (A) Hemolysis images and microscopic morphology of erythrocytes after composite hydrogel incubation. (B) Statistics of hemolysis rate for each group. (C), (E) and (G) live/dead fluorescence images of L929 fibroblasts, HUVECs, and RAW264.7 after hydrogel treatment for 1, 3, and 5 days. (D), (F) and (H) Cell viability of L929 fibroblasts, HUVECs, and RAW264.7 after hydrogel treatment for 1, 3, and 5 days.

Given the intended in vivo use, biocompatibility assessment is crucial [41]. In this study, the cytocompatibility of the hydrogels was evaluated in vitro using live/dead cell staining and the CCK-8 assay. The image in Fig. 4A illustrates the distinct color variations among the four hydrogel groups: the negative control group (PBS) and the positive control group (Triton X-100). All four hydrogel groups displayed a light red hue, comparable to that of the PBS group, whereas the Triton X-100 group appeared bright red. Quantitative data are shown in Fig. 4B, revealing a very low hemolysis ratio in the CEGA group (3.05 %). The addition of EGCG and CM did not significantly increase the hemolysis rate, demonstrating that CEGA hydrogels are non-hemolytic and safe for clinical use.

To further confirm the cytocompatibility of the hydrogels, three cell types, HUVECs, L929 fibroblasts, and RAW264.7, were cultured with extracts of the hydrogels, and cell viability was assessed by live/dead staining analysis on days 1, 3, and 5, respectively. As illustrated in Fig. 4C–E, and G, the number of cells on day 5 was higher than that on day 1, with particularly significant increases in the CGA and CEGA groups. By contrast, only a few dead cells were observed, indicating that the cells grew well throughout the experiment. The CCK-8 assay was used to examine the cell proliferation and viability. On day 5, the CGA and CEGA groups showed significantly enhanced proliferation of all three cell types compared with the other groups (Fig. 4D–F, and H). In conclusion, ASC-CM demonstrated the ability to promote the proliferation of skin-related cells in vitro.

### 3.4 In vitro and in vivo antibacterial assay

**Fig. 5:**
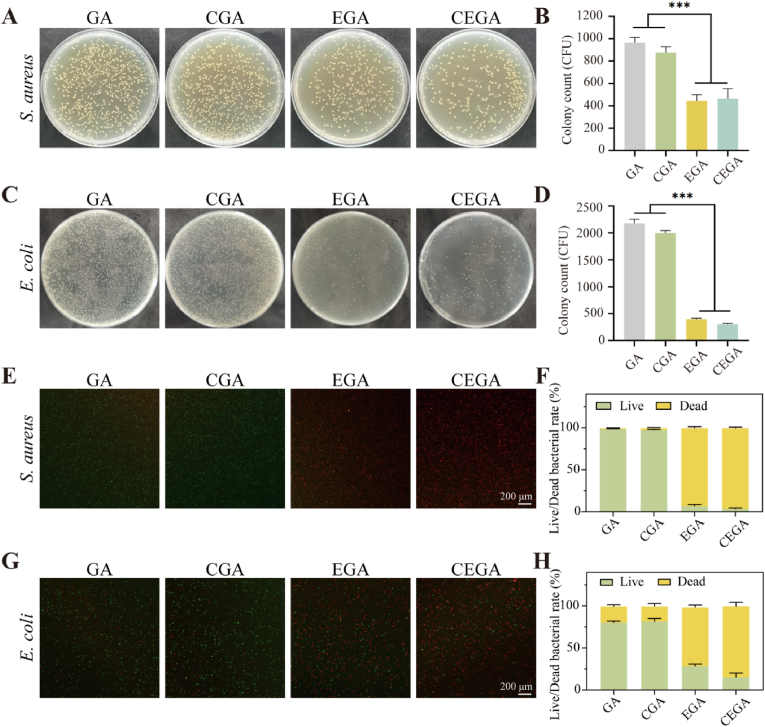
The in vitro antibacterial properties of GA, CGA, EGA, and CEGA hydrogels. (A) Representative colony formation on an LB agar plate for *S. aureus*. (B) Colony counting for *S. aureus*. (C) Representative colony formation on an LB agar plate for *E. coli*. (D) Colony counting for *E. coli*. (E) Live/dead staining image for *S. aureus*. (F) Live/dead bacterial ratio for *S. aureus*. (G) Live/dead staining image for *E. coli*. (H) Live/dead bacterial ratio for *E. coli*.

*S. aureus* and *E. coli* are the two most prevalent bacteria associated with wound infections, posing significant challenges to the healing process. Therefore antibacterial capacity is a critical factor in assessing the function of a wound dressing [42]. Hydrogel's antibacterial properties were evaluated through the spread plate method to determine its impact on bacteria. Different samples were immersed in *S. aureus* or *E. coli* suspensions to measure their contact antibacterial capacities. As shown in Fig. 5A and C, the EGA and CEGA groups showed only small growth of *S. aureus* and *E. coli*, whereas the GA and CGA groups showed significant growth of *S. aureus* and *E. coli*. The results of bacterial colony counts were consistent with the colony growth (Fig. 5B and D). Additionally, the antibacterial properties of the CEGA hydrogel were further confirmed by live/dead bacterial staining of *S. aureus* and *E. coli*. As shown in Fig. 5E and G, both the GA and the CGA groups exhibited distinct green fluorescence (live bacteria), whereas the EGA and CEGA groups predominantly displayed red fluorescence (dead bacteria).

In addition, the in vivo antibacterial assay showed that both the EGA and the CEGA groups exhibited better antibacterial performance than the other three groups on the 1st and 3rd days which was partially consistent with the results of the in vitro antibacterial assay (Fig. S2). These results further validate that the CGA and CEGA hydrogels possess strong antibacterial properties, which aligns with the plate-counting results. These results indicate that the hydrogel groups containing EGCG have superior antibacterial properties and are capable of causing the death of both *S. aureus* and *E. coli*.

### 3.5 Antioxidant activity studies

**Fig. 6:**
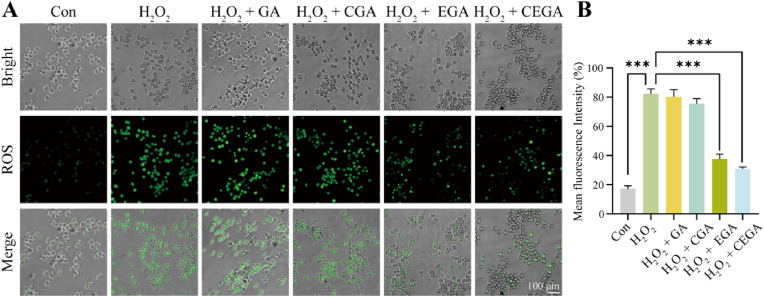
Comparison of reactive oxygen scavenging ability of hydrogels in each group. (A) Fluorescent images of RAW264.7 stained with DCFH-DA. (B) Quantification of DCFH-DA of RAW264.7.

In patients with diabetes, excess ROS resulting from a redox imbalance has been proven to inhibit wound healing through various mechanisms, such as deactivating growth factors and diminishing the production and deposition of the extracellular matrix [43]. To explore whether the CEGA hydrogel can scavenge ROS, a DCFH-DA assay was employed. Fig. 6A and B illustrated that H_2_O_2_ treatment resulted in a greater accumulation of ROS in contrast to untreated cells. Hydrogels with EGCG (EGA and CEGA) reduced ROS production stimulated by hydrogen peroxide. The mean fluorescence intensities of the H_2_O_2_+EGA and H_2_O_2_+CEGA groups were lower than the H_2_O_2_ group. The results revealed that the CEGA hydrogel exhibited robust free radical scavenging properties, on par with the efficacy of EGA. In contrast, GA and CGA exhibited limited capabilities in degrading DPPH free radicals, suggesting that the majority of the scavenging activity was attributed to EGCG.

### 3.6 Migration assay and tube formation assay

Diabetes-induced metabolic disorders impair the proliferation and migration capacities of cells [44]. Moreover, chronic hyperglycemia disrupts glycoprotein synthesis in the endothelial cell basement membrane, leading to increased vascular permeability, which damages the vascular basement membrane and hinders angiogenesis [45]. Therefore, promoting angiogenesis and cell migration are essential for diabetic wound healing. ASCs maintain a rapid growth rate of antlers due to their strong proliferation ability. Previous studies have confirmed that ASC-CM promotes the proliferation and migration of bone marrow stromal cells (BMSCs) and HUVECs in vitro [11].

**Fig. 7:**
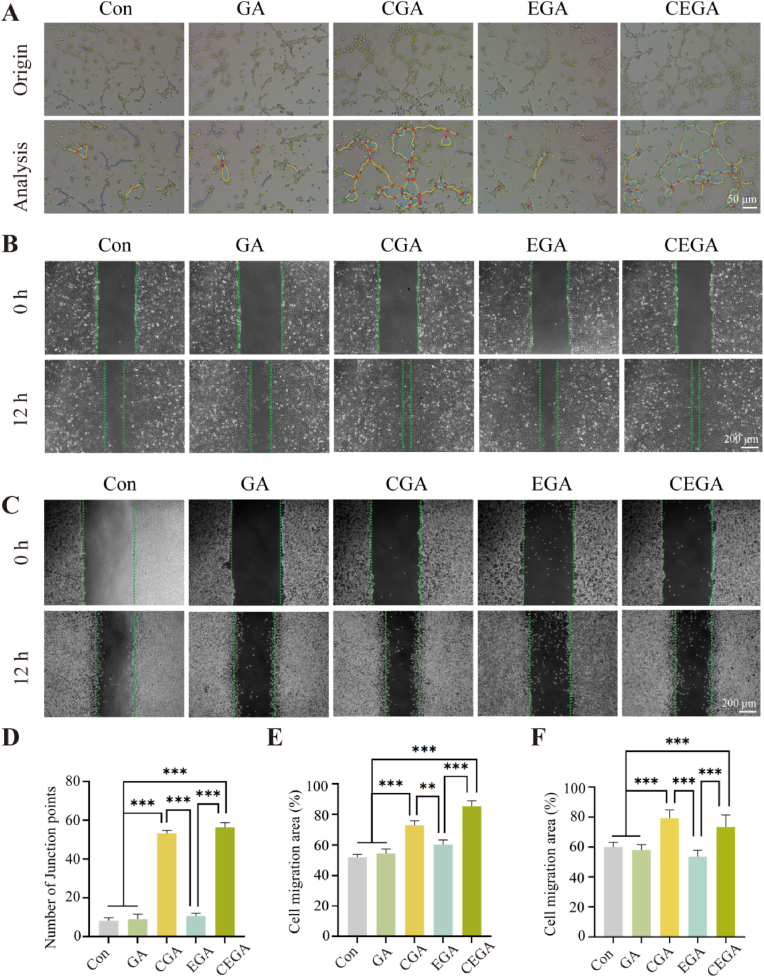
Evaluation of CEGA hydrogel in vitro for enhancing cell migration and angiogenesis. (A) Representative images of tube-forming experiments. (B) Migration abilities of HUVECs after co-culturing with hydrogels. (C) Migration abilities of L929 fibroblasts after co-culturing with hydrogels. (D) Quantitative analysis of the number of junction points. (E) Statistical analysis of HUVECs migration area at 12 h. (F) Statistical analysis of L929 fibroblast migration area at 12 h.

Tube formation and cell scratch assays were performed to evaluate the angiogenic capacity of the study. In the tube formation assay, both the CGA and CEGA groups formed more tubular structures and showed more initial nodes than the Con, GA, and EGA groups (Fig. 7A and D). The migration of HUVECs and L929 fibroblasts was evaluated by scratch assays (Fig. 7B, C, E, and F). The experiment revealed that the wound closure rates of the CGA and CEGA groups were notably elevated at 12 h. To sum up, these findings suggest that the continuous release of ASC-CM by the CEGA hydrogel can promote wound healing through accelerating skin-related cell proliferation and migration.

### 3.7 Macrophage polarization

**Fig. 8:**
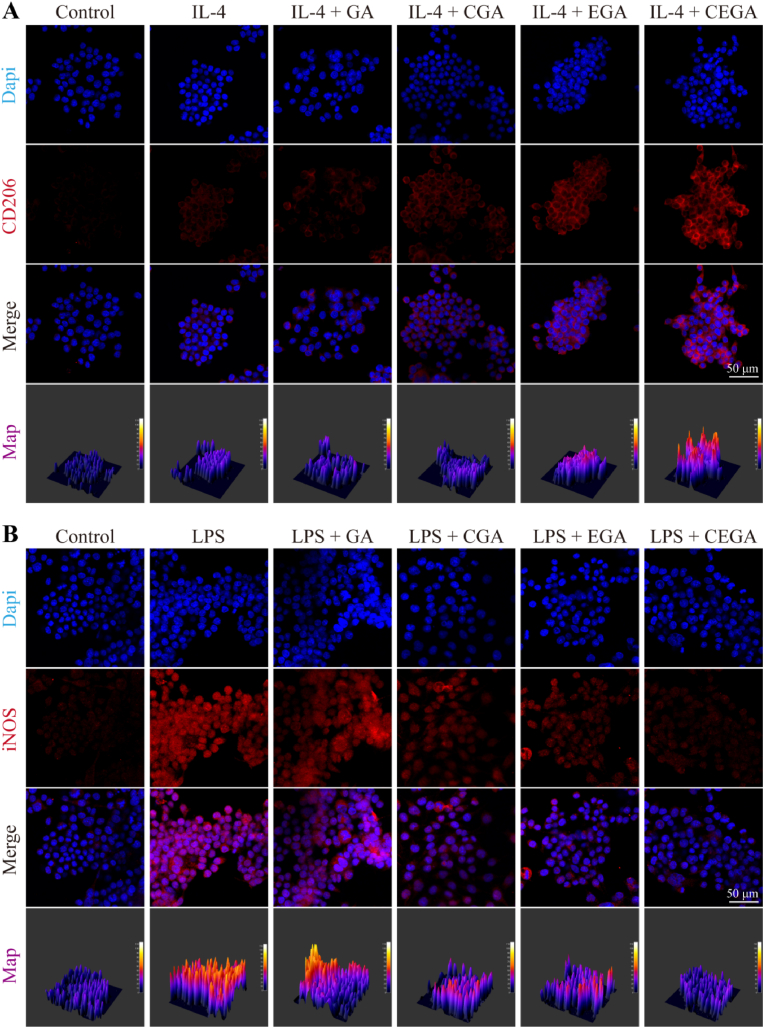
In vitro evaluating immune modulation of CEGA hydrogel. Immunofluorescence images of (A) M2 (CD206) and (B) M1 (iNOS) macrophage marker.

In diabetic wounds, a series of complications arise due to the presence of high glucose levels, which perturbs the normal wound healing process by disrupting the physiological cascade response. This environment makes it extremely challenging for macrophages to polarize from pro-inflammatory (M1) to anti-inflammatory (M2) phenotype [46,47]. The M1 macrophage marker iNOS and M2 macrophage marker CD206 were detected using immunofluorescence staining. As shown in Fig. 8A, the expression of CD206 in the IL-4+CEGA group was the strongest among the groups. Additionally, the quantitative analysis confirmed that the CD206 fluorescence intensity of the RAW264.7 in the IL-4+CEGA group was significantly higher than that in the other groups (Fig. S3A). However, the expression of iNOS in the LPS + CGA, LPS + EGA, and LPS + CEGA groups was lower than that in the LPS and LPS + GA groups (Fig. 8B), and the quantitative analysis were the same. In summary, the CEGA hydrogel effectively facilitates the transition of macrophage phenotype from M1 to M2 and demonstrates enhanced immune regulatory capabilities.

### 3.8 In vivo diabetic wound healing properties of the hydrogels

**Fig. 9:**
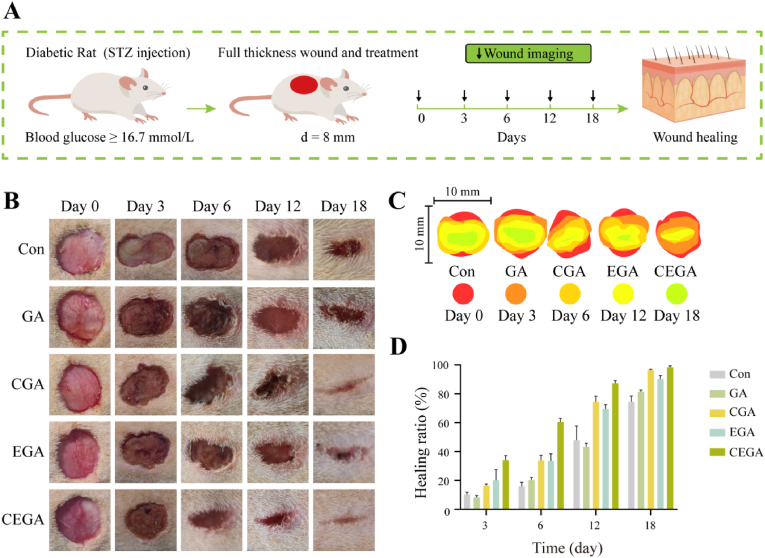
Wound healing assessment in vivo. (A) Schematic diagram of the established diabetic infected wounds and the hydrogel treatment. (B) Photographs of skin wounds of Con, GA, CGA, EGA, and CEGA groups at each indicated time point. (C) Traces of wounds healing with different hydrogel treatments on days 0, 3, 6, 12, and 18. (D) Quantitative analysis of the wound healing rate of each group on days 3, 6, 12, and 18.

To assess the wound healing effectiveness of the hydrogels, the CEGA hydrogel was used to treat full-thickness skin defects in diabetic rats. A circular wound (8 mm in diameter) was created on the backs of the diabetic rats, and the healing process was monitored at 0, 3, 6, 12, and 18 days post-treatment (Fig. 9A). As illustrated in Fig. 9B and C, wounds treated with the CEGA sponge exhibited no erythema or erosion throughout the healing phase, and healing was faster than that in the other groups Additionally, the CGA and EGA groups also demonstrated enhanced wound healing, as shown by the wound area measurements (Fig. 9B and C). By day 12, the healing rate in the CGA group surpassed that of the EGA group, likely because of the high levels of growth factors in ASC-CM that promote the regeneration of new tissues, including epithelial and vascular structures. Fig. 9D demonstrated that, by the 18th post-surgery day, untreated diabetic wounds achieved healing at approximately 74.40 ± 4.18 % of their original size. In contrast, the wound closure percentages in the CGA, EGA, and CEGA hydrogel treatment groups were 96.58 ± 0.43 %, 91.28 ± 2.46 %, and 98.36 ± 1.06 %, respectively. The wounds treated with the CEGA hydrogel exhibited the smallest wound area, suggesting a faster healing process than other groups. In conclusion, the CEGA hydrogel accelerated the wound-healing process.

### 3.9 Histomorphological evaluation

**Fig. 10:**
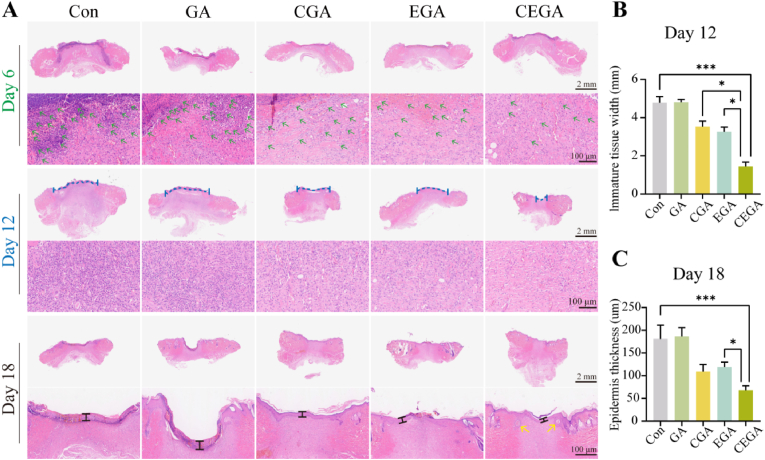
H&E staining analysis in vivo. (A) H&E staining images of wound tissues on days 6, 12, and 18. (B) The dermal space length of wound tissue on day 12. (C) The epidermis thickness of wound tissue on day 18. Green arrows indicate inflammatory cells and yellow arrows indicate skin appendage. (For interpretation of the references to color in this figure legend, the reader is referred to the Web version of this article.)

Histological examination was conducted to assess the quality of the newly formed skin in the defects of the five groups. Observations made during the early stages of wound healing (day 6) revealed a pronounced inflammatory reaction in the Con and GA groups. However, fewer inflammatory cells (green arrow) were found in the wound sites of the CGA/EGA/CEGA group than in those of the other hydrogel groups (Fig. 10A). As shown in Fig. 10B, the Con, GA, CGA, EGA, and CEGA groups remained a gap distance for unrecovered epidermis of 4.78 ± 0.31, 4.81 ± 0.14, 3.53 ± 0.29, 3.26 ± 0.25 and 1.45 ± 0.23 mm on the 12th day, respectively. In contrast, the CEGA group achieved better efficiency of re-epithelialization through H&E staining on day 12. At day 18, the thickness of the epithelium in the CEGA group was 67.98 ± 10.12 μm, which was thinner than the Con group (207.50 ± 30.29 μm), GA group (186.50 ± 19.52 μm), CGA group (113.49 ± 15.33 μm) and EGA group (114.16 ± 10.54 μm) (Fig. 10C). In addition, the regenerated skin in the CEGA group showed better tissue structure than that in the other groups, with more regenerated skin appendages (yellow arrows) in the subcutaneous tissue, suggesting the best re-epithelialization effect. These results demonstrate that the CEGA hydrogel can reduce the size of wounds and inhibit inflammatory reactions.

**Fig. 11:**
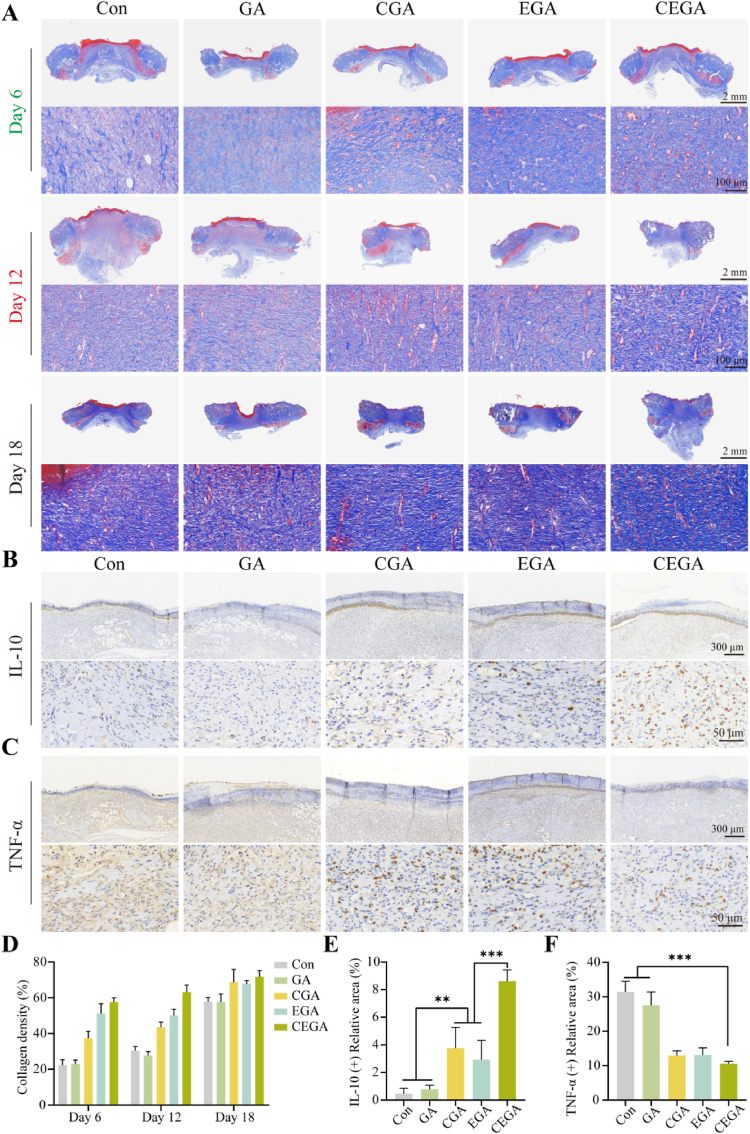
Histopathologic analysis in vivo. (A) Masson staining images of wound tissues on days 6, 12, and 18. Immunohistochemistry staining of wound tissue of (B) IL-10 and (C) TNF-α. (D) The collagen density of wound tissue on days 6, 12 and 18. Quantitative analysis of (E) IL-10, (F) TNF-α.

We further observed collagen deposition using histological analysis. As shown in Fig. 11A, after 6 days, the Con, GA, and EGA groups showed less collagen deposition and loose and sparse collagen distribution. In contrast, the CGA and CEGA hydrogel groups exhibited increased collagen deposition. After 18 days, as shown in Fig. 10B, in the CEGA group, fibroblasts were proliferating under the epidermis, and collagen deposition was sufficient and orderly. These results suggest that CEGA hydrogel can inhibit inflammatory responses, reduce the size of wounds, and promote re-epithelialization.

Persistent inflammation is a significant contributor to delayed wound healing in diabetic rats. TNF-α is extensively expressed during inflammation, and as the degree of inflammation diminishes, there is an increase in the expression of IL-10, whereas the levels of TNF-α decline [48]. Therefore, to further evaluate the mechanism of the hydrogel promoting wound healing on day 6, IL-10 and TNF-α were chosen as biomarkers to assess the inflammatory response during the wound healing process of wound dressings. Immunohistochemical staining of IL-10 (Fig. 11B) and the quantitative results (Fig. 11E) illustrated that the CEGA hydrogel group showed the highest IL-10 expression compared with other groups. On the contrary, the expression level of TNF-α in the CEGA hydrogel group is the lowest (Fig. 11C and F). These results suggested that CEGA hydrogel can augment the synthesis of IL-10 and mitigate the levels of TNF-α during the initial phase of wound tissue regeneration, thereby inhibiting inflammation.

### 3.10 In vivo immunomodulatory and angiogenic capacities

**Fig. 12:**
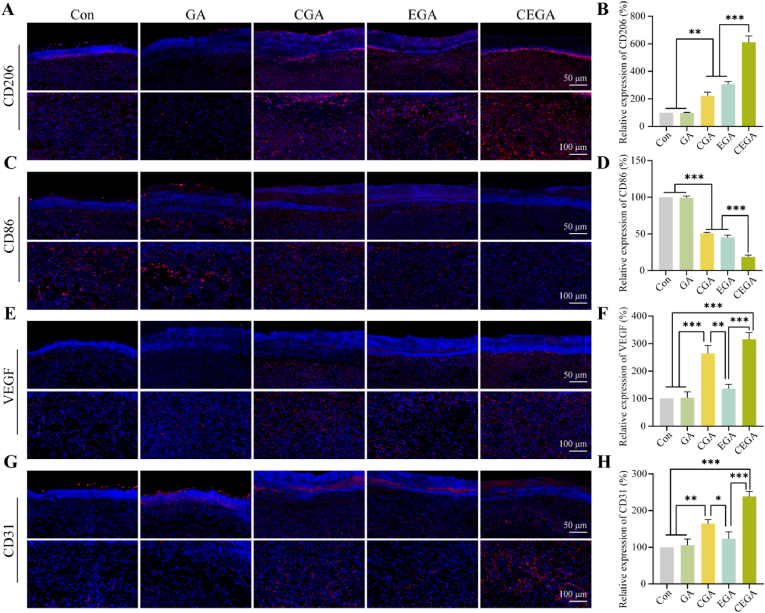
Immunomodulatory, and pro-angiogenesis properties of hydrogels in diabetic wounds. Immunofluorescence staining of wound tissue of (A) CD206, (C) CD86 (E) VEGF and (G) CD31 on day 6. Quantitative analysis of (B) CD206, (D) CD86, (F) VEGF, and (H) CD31 on day 6.

Based on the in vitro experimental results, we further investigated whether the reduction in the inflammatory response and improvement in the healing of diabetic wounds treated with CEGA hydrogels were associated with the polarization state of macrophages in the wound. Protein levels of M1 macrophage labeled CD86 and M2 macrophage labeled CD206 were detected using immunofluorescence staining. As shown in Fig. 12A and C, on the 6th day, the expression level of CD206 was the highest in the CEGA group, and the expression level of CD86 was the lowest, which was consistent with the quantitative analysis results (Fig. 12B and D). These results indicate that the CEGA hydrogel can transform pro-inflammatory M1 macrophages into anti-inflammatory M2 macrophages.

During the proliferation phase of wound healing, the emergence of neovascularization is vital for facilitating the delivery of nutrients and oxygen to the wound site, thereby supporting fibroblast growth, collagen production, and re-epithelialization [49]. In this study, we labeled VEGF and CD31 (a marker of the neovascular endothelium) using an immunofluorescence assay to investigate the promotional effect of CEGA hydrogels on angiogenesis. VEGF is an essential effector in the late inflammatory and reparative-proliferative phases of wound inflammation and is involved in wound granulation tissue formation, vascular proliferation, and collagen fiber synthesis [50]. In Fig. 12E and F, it can be seen that the expression of VEGF in the CGA and CEGA groups was much higher than that in the Con, GA, and EGA groups for 6 days. CD31, a heavily glycosylated Ig-like membrane receptor, is predominantly expressed in the vascular endothelial cells and is particularly concentrated near wounds [51,52]. As shown in Fig. 12G and H, the CGA and CEGA hydrogels appeared to have grossly increased expression of CD31 compared to the other groups on the 6th day. In summary, the CEGA hydrogel can stimulate vascular regeneration, which is also consistent with the results of in vitro tube formation assays.

## 4 Conclusion

In this study, we constructed a dual-network hydrogel (CEGA) capable of promoting cell proliferation, angiogenesis, antibacterial activity, anti-inflammatory responses, and the regulation of macrophage polarization for diabetic wound treatment. The CEGA hydrogel effectively retained and delivered multiple bioactive factors from ASC-CM, which were demonstrated to play roles in cell proliferation, angiogenesis, and macrophage polarization. In addition, EGCG has antibacterial and antioxidant properties. The CEGA hydrogel, which releases EGCG, responds to bacterial infections and protects cells from oxidative stress damage. These findings indicated its potential as a novel dressing for diabetic skin wounds. These results provide a solid foundation for the clinical application of this hydrogel in wound treatment and offer promising prospects for incorporating various drugs and active protein factors into this hydrogel in future applications.

## CRediT authorship contribution statement

**Xianyi He:** Writing – original draft, Validation, Software, Methodology, Formal analysis, Data curation, Conceptualization. **Yichen Gao:** Validation, Methodology, Formal analysis, Data curation. **Xia Wang:** Methodology, Formal analysis. **Chuankai Zhang:** Visualization, Software. **Zhaoxin Xia:** Software, Resources. **Wei Xu:** Validation, Investigation. **Hua Yang:** Software, Investigation. **Gang Tao:** Writing – review & editing, Formal analysis. **Rui Cai:** Supervision, Methodology, Investigation. **Junliang Chen:** Writing – review & editing, Visualization, Funding acquisition, Formal analysis, Data curation. **Yun He:** Writing – review & editing, Visualization, Methodology, Investigation, Funding acquisition, Data curation.

## Ethics approval and consent to participate

All animal experiments were approved by the animal ethics committee of Southwest Medical University (NO.: 20211117-003).

## Declaration of competing interest

The authors declare that they have no known competing financial interests or personal relationships that could have appeared to influence the work reported in this paper.

## Data Availability

The data that support the findings of this study are available from the corresponding author upon reasonable request.
