# Supplementary material for: Dual-network hydrogel loaded with antler stem cells conditioned medium and EGCG promotes diabetic wound healing through antibacterial, antioxidant, anti-inflammatory, and angiogenesis
Source: Mater Today Bio. 2025 Feb 24;31:101612. doi: 10.1016/j.mtbio.2025.101612 (PMC11914521; doi:10.1016/j.mtbio.2025.101612)
Supplement: Multimedia component 1 [file mmc1.docx]

**Dual-network hydrogel loaded with antler stem cells conditioned medium and EGCG promotes diabetic wound healing through antibacterial, antioxidant, anti-inflammatory, and angiogenesis**


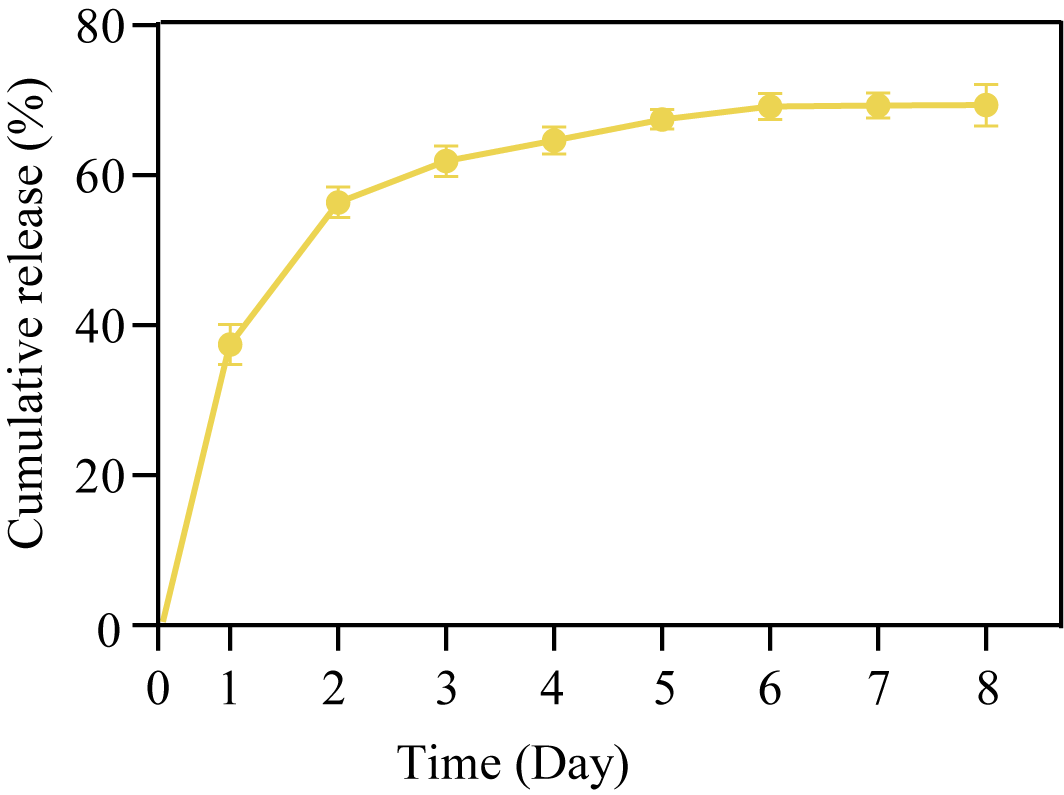


**Fig. S1.** Cumulative release properties of CEGA hydrogel for ASC-CM.


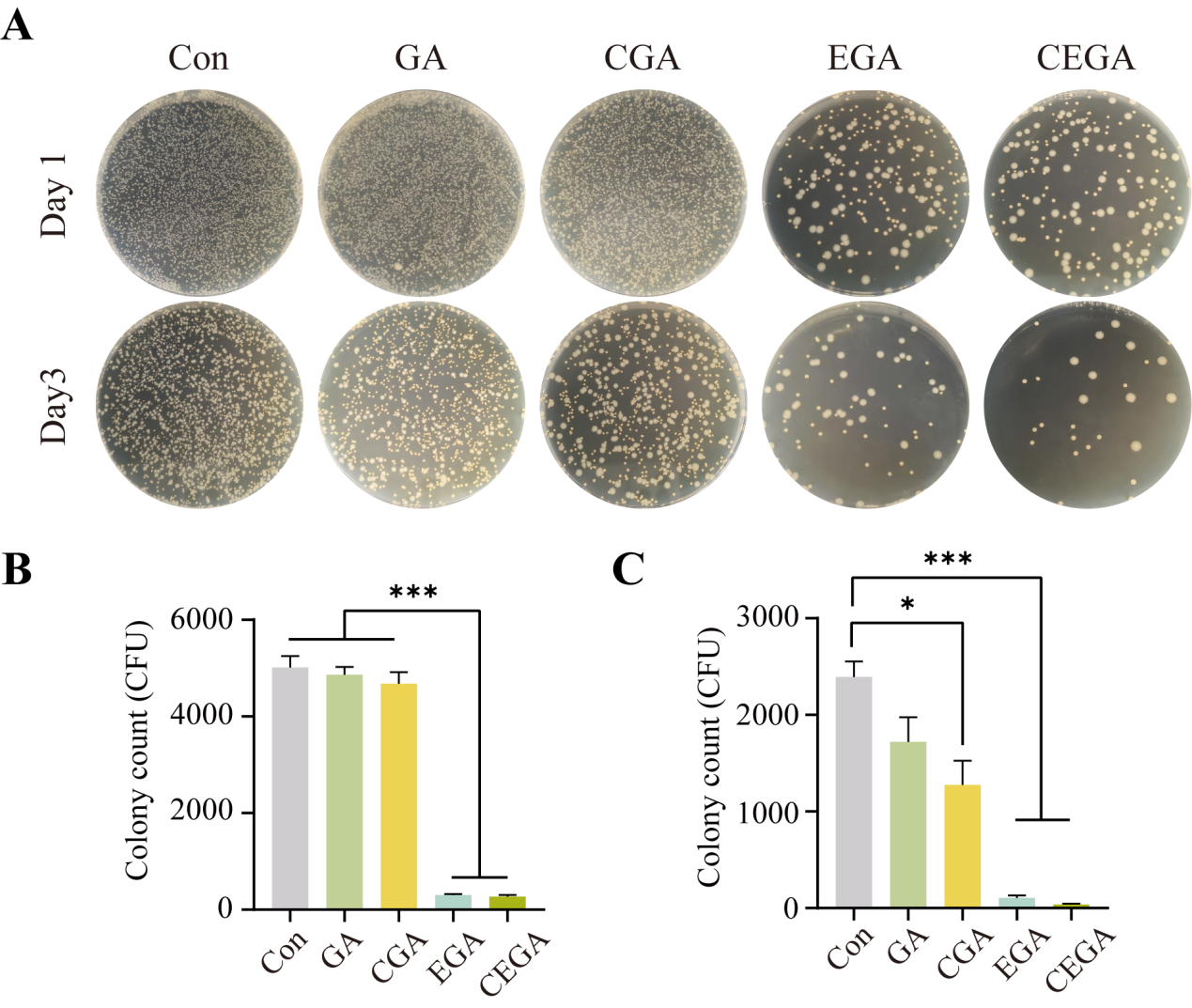


**Fig. S2.** The in vivo antibacterial properties of GA, CGA, EGA, and CEGA hydrogels. (A) The antibacterial activity of the hydrogels in the wound healing on day 1 and 3. (B) Colony counting on day 1. (C) Colony counting on day 3.


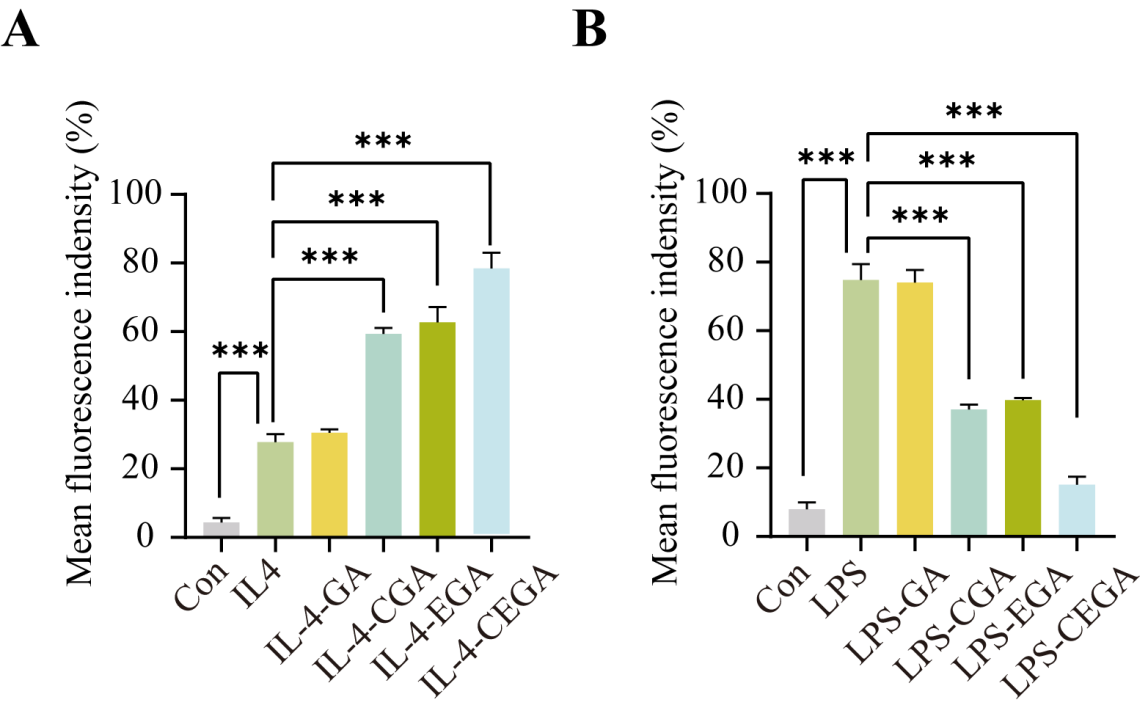


**Fig. S3.** In vitro evaluating immune modulation of CEGA hydrogel. Quantitative analysis of immunofluorescence of (A) M2 (CD206) and (B) M1 (iNOS) macrophage marker.
